# A Tool to Quantify the Functional Impact of Oscillopsia

**DOI:** 10.3389/fneur.2018.00142

**Published:** 2018-03-15

**Authors:** Eric R. Anson, Yoav Gimmon, Tim Kiemel, John J. Jeka, John P. Carey

**Affiliations:** ^1^Department of Otolaryngology Head and Neck Surgery and the David M. Rubinstein Hearing Center, Johns Hopkins Medical Institutes, Johns Hopkins University School of Medicine, Baltimore, MD, United States; ^2^Department of Otolaryngology, University of Rochester, Rochester, NY, United States; ^3^Laboratory of Vestibular NeuroAdaptation, Department of Otolaryngology – Head and Neck Surgery, Johns Hopkins University School of Medicine, Baltimore, MD, United States; ^4^Kinesiology Department, University of Maryland, College Park, College Park, MD, United States; ^5^Kinesiology Department, University of Delaware, Newark, DE, United States

**Keywords:** oscillospia, vestibular loss, activity and participation restriction, balance, mobility

## Abstract

**Background:**

Individuals with bilateral vestibular hypofunction (BVH) often report symptoms of oscillopsia during walking. Existing assessments of oscillopsia are limited to descriptions of severity and symptom frequency, neither of which provides a description of functional limitations attributed to oscillopsia. A novel questionnaire, the Oscillopsia Functional Impact scale (OFI) was developed to describe the impact of oscillopsia on daily life activities. Questions on the OFI ask how often individuals are able to execute specific activities considered to depend on gaze stability in an effort to link functional mobility impairments to oscillopsia for individuals with vestibular loss.

**Methods:**

Subjective reports of oscillopsia and balance confidence were recorded for 21 individuals with BVH and 48 healthy controls. Spearman correlation coefficients were calculated to determine the relationship between the OFI and oscillopsia visual analog scale (OS VAS), oscillopsia severity questionnaire (OSQ), and Activities-Specific Balance Confidence scale to demonstrate face validity. Chronbach’s α was calculated to determine internal validity for the items of the OFI. A one-way MANOVA was conducted with planned *post hoc* paired *t*-tests for group differences on all oscillopsia questionnaires using a corrected α = 0.0125.

**Results:**

The OFI was highly correlated with measures of oscillopsia severity (OS VAS; *r* = 0.69, *p* < 0.001) and frequency (OSQ; *r* = 0.84, *p* < 0.001) and also with the Activities-Specific Balance Confidence scale (*r* = −0.84, *p* < 0.001). Cronbach’s α for the OFI was 0.97. Individuals with BVH scored worse on all measures of oscillopsia and balance confidence compared to healthy individuals (*p*’s < 0.001).

**Conclusion:**

The OFI appears to capture the construct of oscillopsia in the context of functional mobility. Combining with oscillopsia metrics that quantify severity and frequency allows for a more complete characterization of the impact of oscillopsia on an individual’s daily behavior. The OFI discriminated individuals with BVH from healthy individuals.

## Introduction

During locomotion, the ability to see clearly in order to avoid or interact with objects or to facilitate use of optic flow for balance and heading is essential (1, 2). The primary purpose of the vestibulo-ocular reflex (VOR) may be to stabilize gaze during locomotion, when frequencies of head movement far exceed the capabilities of other eye movement systems (3, 4). Gaze instability during walking has been directly attributed to loss of function of the VOR (5–9). Impaired gaze stabilization makes navigation and obstacle avoidance during walking more challenging, which may contribute to gait variability in individuals with bilateral vestibular hypofunction (BVH) (10). After VOR failure, a commonly reported complaint was that stationary environmental objects appear to “jump” during walking (6). Oscillopsia has also been reported in individuals with intact saccular function further indicating that oscillopsia symptoms may depend on angular VOR capabilities (11). However, complaints of oscillopsia are not consistent across all individuals with a diagnosis of BVH (12).

Current physiological vestibular function tests do not adequately characterize oscillopsia or the daily life impairments experienced by individuals with BVH. Oscillopsia has been studied using visual analog scales of symptom severity (5, 13) or symptom frequency (9, 14). However, oscillopsia severity and frequency do not consistently relate to physiological (i.e., VOR) or perceptual assessments of vestibular function like dynamic visual acuity (DVA) (5, 9, 12, 15–17). This disconnect between diagnostic testing and subjective quality of life may represent a limitation in the ability of existing questionnaires or diagnostic tests to adequately capture the functional impact of oscillopsia symptoms on daily life. Combining physiological assessments of gaze stability by simultaneous measurement of the VOR and other oculomotor responses and reading capability using tests such as the HITD will help to close the gap between physiology and function (18), but may not fully account for reported symptoms of oscillopsia. Recently, the presence of oscillopsia symptoms in individuals with BVH was found to correlate with their performance on a suppression head impulse test (SHIMP) (19). There are no self-report symptom scales that specifically characterize the impact of oscillopsia symptoms on the ability to execute daily life activities. This suggests that subjective measures that are linked to functional daily life tasks are needed to more completely describe the relationship between vestibular pathology and oscillopsia and the impact of both on an individual.

The two most common subjective measures of oscillopsia are the oscillopsia visual analog scale [OS VAS (13)] and an oscillopsia severity questionnaire [OSQ (9)]. The OS VAS describes oscillopsia symptom severity and the OSQ describes symptom frequency but is not specific to head motion-induced oscillopsia. Although very important in characterizing disease state, symptom severity and frequency may not adequately characterize the ability to execute daily life activities, which could explain the inconsistent relationship between VOR gain, DVA scores, and oscillopsia symptoms. The existing scales do not adequately characterize how oscillopsia impacts daily function from an activity or participation perspective as described by the International Classification of Functioning, Disability, and Health (ICF) (20). The ICF model includes four domains: (1) body functions; (2) body structures; (3) activities and participation; and (4) environmental factors. The WHO defines activities as “the execution of a task or action by an individual” and participation as “involvement in a life situation” (20). The OS VAS and OSQ would both address the Body Functions domain of the ICF as would diagnostic measures of vestibular function like VOR gain and caloric responses. Imaging and postoperative anatomical status would provide information in the domain of Body Structures. There are currently no *oscillopsia specific* measures, which address the ICF domain of Activities and Participation (15). Oscillopsia has been reported to diminish quality of life *via* activity restriction (21, 22); therefore, development of a valid scale that can identify the impact of oscillopsia on the ability to perform or participate in specific activities is greatly needed.

A new questionnaire, the Oscillopsia Functional Impact (OFI) scale (see Supplementary Material) was developed to more completely characterize the impact of oscillopsia on daily life. The OFI was designed to characterize the impact of oscillopsia during functional mobility and other tasks with implicit visual acuity or visual attention components for individuals with vestibular loss. We investigated whether the OFI had face validity based on existing scales related to oscillopsia and balance ability and whether the OFI could discriminate between healthy individuals and individuals with vestibular hypofunction.

## Materials and Methods

### Subjects

Sixty nine individuals (33 males, 36 females) participated in this experiment after providing informed consent. A diagnosis of BVH was made based on weak (<10°/s combined per ear) or absent caloric responses and/or bilaterally pathologic head impulse tests (23, 24). Healthy individuals did not have a history of vertigo, dizziness, or balance problems. 48 healthy individuals and 21 individuals with BVH participated in the study. This study was carried out in accordance with the recommendations of the institutional review boards at Johns Hopkins School of Medicine and the University of Maryland, and all subjects gave written informed consent in accordance with the Declaration of Helsinki. The protocol was approved by the institutional review boards at Johns Hopkins School of Medicine and the University of Maryland.

### Procedures

Each subject completed several questionnaires including: (1) the Activity-Specific Balance Confidence scale (ABC) (25); (2) the oscillopsia visual analog scale (OS VAS) (13); (3) the oscillopsia severity questionnaire (OSQ) (9); and (4) the Oscillopsia Functional Impact (OFI) scale developed for this experiment. Questions on the OFI were designed to identify the degree to which oscillopsia interferes with execution of daily activities based on subjective complaints reported by patients seen by John P. Carey and Eric R. Anson in their respective clinical practice. The OFI was modeled after a scale designed to characterize symptoms of autophony for individuals with superior canal dehiscence (26) and is scored out of a total of 215 points with a maximum score of 5 for each question with “n/a” scored as a 0. Individuals were instructed to use the following scale to answer each question on the OFI: Not at all, A little of the time, Some of the time, A good deal of the time, Almost all the time, I have given up this activity because of symptoms, Don’t know, as I just don’t do this activity. Questions 12, 13, 14, 15, 16, 21, 22, 23, and 24 were phrased negatively and are scored in reverse order.

### Data Analysis

Cronbach’s α was calculated to determine internal consistency for the OFI scores. To determine face validity, Spearman’s correlation coefficients were calculated to determine the relationships between OFI total scores, OS VAS scores, OSC scores, and ABC scores. A *t*-test was used to determine whether there was a difference in age between groups. A one-way MANOVA was conducted with planned *post hoc t*-tests for group differences on all questionnaires. Significance was tested at α = 0.05 for Cronbach’s α and Spearman’s correlation and a Bonferroni corrected α = 0.0125 was used for *post hoc t*-tests.

## Results

One healthy individual declined to provide his age. The mean age for the rest of the healthy individuals was 44.1 [range (19–75); SD = 18.1] and the mean age for the individuals with BVH was 60.84 [range (35–80); SD = 13.0]. The control group was significantly younger than the BVH group (*t* = −3.8086, *p* = 0.002).

Cronbach’s α for OFI scores was 0.97 demonstrating high internal consistency for the individual items of the OFI. The OFI scores were highly correlated with the other subjective measures of oscillopsia and balance confidence, see Table 1. The MANOVA was highly significant for between group differences on the distribution of scores on all questionnaires [*F*(4, 64) = 36.5, *p* < 0.001]. Subsequent *post hoc* group comparisons are as follows. Individuals with BVH reported greater oscillopsia severity [*t*(2, 67) = 11.69, *p* < 0.001], higher oscillopsia frequency [*t*(2, 67) = 9.91, *p* < 0.001], greater impact of oscillopsia on activity performance [*t*(2, 67) = 9.62, *p* < 0.001], and lower balance confidence [*t*(2, 67) = −10.31, *p* < 0.001] compared to healthy individuals. Group average scores and 95% confidence intervals for each of the questionnaires are presented in Table 2.

**Table 1:** Spearman correlation coefficients between subjective rating scales of oscillopsia and balance confidence.

	OS VAS	OSC	ABC scale
OFI total	0.69*	0.84*	−0.84*
	*p* < 0.001	*p* < 0.001	*p* < 0.001
OS VAS		0.77*	−0.69*
		*p* < 0.001	*p* < 0.001
OSC			−0.84*
			*p* < 0.001

Significant correlations are indicated with an *.

*ABC scale, Activity-Specific Balance Confidence scale; OFI, Oscillopsia Functional Impact scale; OSC, Oscillopsia Severity scale; OS VAS, Oscillopsia Visual Analog Scale*.

**Table 2:** Between group differences on subjective measures of oscillopsia and balance.

Group	OFI total	OSC	OS VAS	ABC scale
Healthy	12.0 (1.6)	1.2 (0.04)	0.1 (0.03)	96.0 (0.67)
	[8.8–15.3]	[1.10–1.26]	[0.03–0.16]	[94.7–97.4]
BVH	65.9 (7.7)*	2.7 (0.22)*	5.0 (0.63)*	67.4 (4.0)*
	[49.8–81.9]	[2.3–3.2]	[3.6–6.3]	[59.2–75.6]

Average (SEM) [95% CI] scores are presented and significant differences from healthy individuals are indicated by an *, all *p* < 0.001.

*ABC scale, Activity-Specific Balance Confidence scale; BVH, bilateral vestibular hypofunction; OFI, Oscillopsia Functional Impact scale; OSC, Oscillopsia Severity scale; OS VAS, Oscillopsia Visual Analog Scale*.

Because the control group was significantly younger than the individuals with BVH, we performed a sensitivity analysis by repeating the MANOVA excluding all subjects under 60 years old. The overall sample was reduced to 16 healthy controls and 12 individuals with BVH. The statistical results were the same. The MANOVA was highly significant for between group differences on the distribution of scores on all questionnaires [*F*(4, 23) = 17.6, *p* < 0.001]. Subsequent *post hoc* group comparisons are as follows. Individuals with BVH reported greater oscillopsia severity [*t*(2, 26) = 8.04, *p* < 0.001], higher oscillopsia frequency [*t*(2, 26) = 5.89, *p* < 0.001], greater impact of oscillopsia on activity performance [*t*(2, 26) = 5.68, *p* < 0.001], and lower balance confidence [*t*(2, 67) = −8.01, *p* < 0.001] compared to healthy individuals.

## Discussion

Overall, the OFI demonstrated high internal consistency as well as excellent face validity based on high correlations with other measures of oscillopsia and also the ABC scale. Scores on the OFI and OS VAS and OSQ were all positively correlated indicating that oscillopsia related activity restriction, oscillopsia severity, and oscillopsia frequency all increase together. The strong relationship between the OFI and the ABC scale indicates that the OFI captured limitations in execution of daily life activities for the individuals with vestibular loss. OFI scores appear to capture activity restriction due to oscillopsia symptoms; however, the cause of the activity restriction remains to be determined. The individuals with BVH may be limited in their ability to perform the specific tasks due to oscillopsia from gaze instability. However, individuals with BVH often also present with gait and balance impairments that may contribute to changes in activity independent of oscillopsia. Additionally, cognitive or emotional factors may result in self-imposed participation restriction, with limited involvement in life situations. Future work is needed to determine whether oscillopsia independently contributes to limitations in task performance or participation in daily life.

Individuals with BVH reported more severe oscillopsia (OS VAS), more frequent episodes of oscillopsia (OSQ), and greater functional impairment (OFI) compared to healthy individuals, even when the sample was restricted to individuals 60 years and older. The present results extend those prior results regarding symptom frequency (OSQ) to symptom severity (OS VAS) and activity restriction (OFI). The average OSQ score for individuals with BVH in this cohort was only 2.6 which is lower than that reported previously (9). The difference in OSQ scores for individuals with BVH between studies highlights the variable nature of subjective reports across diagnoses of BVH. Some individuals in this cohort may have greater tolerance for oscillopsia, more residual vestibular function, or more completely developed adaptive anticipatory behavior following the vestibular loss.

Inconsistent oscillopsia complaints have been attributed to learned anticipatory mechanisms (27), which would include feed-forward saccades that occur during head motion (28). It is also possible that due to limitations in vestibular diagnostic testing, individuals diagnosed with BVH may have substantial residual vestibular function (11, 23). Tolerance for perceived retinal image motion has also been proposed as an explanation for the discordance between oscillopsia symptoms (whether severity, frequency, or participation based) and VOR gain or DVA (14). Recently, using a SHIMP paradigm allowed identification of two distinct saccadic responses in individuals with vestibular loss (19). A subset of individuals with vestibular loss demonstrated consistent covert saccades that would be compensatory for a deficient VOR, and subsequent saccades to shift gaze back to the visual target, which had moved. This suggests that a pre-planned covert saccade may result in more optimal gaze stability; however, the impact on oscillopsia symptoms remains to be investigated.

Activity restriction, as measured by the OFI, may depend on multiple factors. Individuals with vestibular loss are known to have balance impairments attributable to the vestibular pathology (29, 30). Reduced vestibular afference will impact both the VOR and vestibulo-spinal reflex pathways. It is possible that scores on some of the items in the OFI are impacted by changes in balance rather than gaze instability and resulting oscillopsia symptoms. In an effort to minimize this confound, the OFI items related to balancing behaviors are also tied to the presence of oscillopsia symptoms or a gaze target task. Additionally, belief (or fear) that secondary symptoms (i.e., falls, dizziness, oscillopsia, anxiety/depression) will result in negative effects (injury, embarrassment) may contribute to activity restriction in ways not identified here (31, 32). Many of the context-based questions involve dual tasking (like walking and texting or reading) and brain fog or mental fatigue could result in activity restriction more related to cognitive or attentional resources and less related to oscillopsia (33). Future studies should investigate this in cohorts of individuals with chronic brain fog and non-vestibular oscillopsia.

### Limitations

The OFI as described here is lengthy and a shorter version would enhance clinical utility. Some of the questions may characterize similar constructs, and future work is needed to examine whether a shorter version of the OFI would have similar validity and discriminatory ability while characterizing the impact of unstable vision on activity restriction. The results presented here may not generalize to less severe presentations BVH such as may occur with aging. Test retest reliability and change over time will need to be established to enhance clinical utility.

## Conclusion

The OFI captures the construct of oscillopsia in the context of mobility and activity restriction. Combining the OFI with existing oscillopsia metrics that quantify severity and frequency allows for a more complete characterization of the impact of oscillopsia symptoms.

## Ethics Statement

This study was carried out in accordance with the recommendations of the institutional review boards at Johns Hopkins School of Medicine and the University of Maryland and all subjects gave written informed consent in accordance with the Declaration of Helsinki. The protocol was approved by the institutional review boards at Johns Hopkins School of Medicine and the University of Maryland.

## Author Notes

Recruitment of additional subjects at the request of the reviewers necessitated the addition of an additional author YG who collected and analyzed data for those subjects.

## Author Contributions

EA and JC developed the OFI. EA, TK, JJ, and JC conceived of and designed the study. EA and YG collected these data. EA drafted the manuscript. TK, JC, JJ, and YG critically edited the manuscript.

## Conflict of Interest Statement

The authors declare that the research was conducted in the absence of any commercial or financial relationships that could be construed as a potential conflict of interest.
