# Supplementary material for: A Tool to Quantify the Functional Impact of Oscillopsia
Source: Front Neurol. 2018 Mar 15;9:142. doi: 10.3389/fneur.2018.00142 (PMC5862789; doi:10.3389/fneur.2018.00142)
Supplement: Supplementary file 1 [file Data_Sheet_1.PDF]

frontiers

1 Supplemental Material

Appendix A. Oscillopsia Functional Index

Oscillopsia Functional Impact Scale

For each of the following questions, select the answer that best describes how often or how severe the indicated symptom affects you in your daily life. Please use the following scale and answer each question.

0 = Not at all

1 = A little of the time

2 = Some of the time

3 = A good deal of the time

4 = Almost all the time

5 = I have given up this activity because of symptoms

n/a = Don’t know, as I just don’t do this activity

		Not at all	A little of the time	Some of the time	A good deal of the time	Almost all the time	I have given up this activity because of symptoms	Don’t know, as I just don’t do this activity
1.	How often does the world around you seem to move / bounce / jump when you are sitting still?	0	1	2	3	4	5	n/a
2.	How often does the world around you seem to move / bounce / jump when you are standing still?	0	1	2	3	4	5	n/a
3.	How often does the world around you seem to move / bounce / jump when you are walking?	0	1	2	3	4	5	n/a

		Not at all	A little of the time	Some of the time	A good deal of the time	Almost all the time	I have given up this activity because of symptoms	Don't know, as I just don't do this activity
4.	How often does the world around you seem to move / bounce / jump when you are running?	0	1	2	3	4	5	n/a
5.	How often does the world around you seem to move / bounce / jump when driving a car?	0	1	2	3	4	5	n/a
6.	How often does the world around you seem to move / bounce / jump when riding in a car?	0	1	2	3	4	5	n/a
7.	How often do you have trouble finding food items you are looking for when grocery shopping?	0	1	2	3	4	5	n/a
8.	How often do you miss a turn when driving somewhere new because you could not read the sign?	0	1	2	3	4	5	n/a
9.	How often do you miss a turn when walking somewhere new because you could not read a sign?	0	1	2	3	4	5	n/a
10.	How often do you have difficulty recognizing familiar faces as you approach a group of people?	0	1	2	3	4	5	n/a
11.	How often do you let other people drive because you might miss a turn?	0	1	2	3	4	5	n/a
12.	How often are you able to use your mobile phone while walking to make a call?	0	1	2	3	4	5	n/a
13.	How often are you able to use your mobile phone while walking to **send** a text / email?	0	1	2	3	4	5	n/a
14.	How often are you able to use your mobile phone while walking to **read** a text / email?	0	1	2	3	4	5	n/a
15.	How often are you able to use your mobile phone while a passenger in a car to **send** a text / email?	0	1	2	3	4	5	n/a
16.	How often are you able to use your mobile phone while a passenger in a car to **read** a text / email?	0	1	2	3	4	5	n/a
17.	How often do you have to stop walking to use your mobile phone to make a call?	0	1	2	3	4	5	n/a

		Not at all	A little of the time	Some of the time	A good deal of the time	Almost all the time	I have given up this activity because of symptoms	Don't know, as I just don't do this activity
18.	How often do you have to take extra time when crossing a street / walking in a parking lot to check for cars because it is difficult to tell when the cars are moving?	0	1	2	3	4	5	n/a
19.	How often do you avoid spending time with family / friends because the world around you seems to move / bounce / jump?	0	1	2	3	4	5	n/a
20.	How often does it take you extra time to find a specific book / movie on a shelf at the store?	0	1	2	3	4	5	n/a
21.	How often are you able to read a shopping list while you are walking at your normal speed?	0	1	2	3	4	5	n/a
22.	How often are you able to read a shopping list while you are walking at a **slower** than normal speed?	0	1	2	3	4	5	n/a
23.	How often are you able to read a shopping list while you are walking at a **faster** than normal speed?	0	1	2	3	4	5	n/a
24.	How often are you able to read a shopping list while you push a cart and walking at normal speed?	0	1	2	3	4	5	n/a
25.	How often do you feel isolated because the world around you seems to move / bounce / jump?	0	1	2	3	4	5	n/a
26.	How often do you feel out of control because the world around you seems to move / bounce / jump?	0	1	2	3	4	5	n/a
27.	How often do you fall down because the world around you seems to move / bounce / jump?	0	1	2	3	4	5	n/a
28.	How often do you trip without falling down because the world around you seems to move / bounce / jump?	0	1	2	3	4	5	n/a
29.	How often do you avoid using stairs because the world around you seems to move / bounce / jump?	0	1	2	3	4	5	n/a
30.	How often do you have to stop walking to read your watch to find out what time it is?	0	1	2	3	4	5	n/a

		Not at all	A little of the time	Some of the time	A good deal of the time	Almost all the time	I have given up this activity because of symptoms	Don't know, as I just don't do this activity
31.	How often does the world around you seem to move / bounce / jump more when you walk on grass or sand?	0	1	2	3	4	5	n/a
32.	How often have you stopped participating in recreational activities because the world around you seems to move / bounce / jump?	0	1	2	3	4	5	n/a
33.	How often have you changed jobs / had difficulty maintaining a job because the world around you seems to move / bounce / jump?	0	1	2	3	4	5	n/a
34.	How often have you avoided driving because the world around you seems to move / bounce / jump?	0	1	2	3	4	5	n/a
35.	If you drop a ball (or other object) that starts to roll away from you, how often do you wait for it to stop moving before you move to pick it up?	0	1	2	3	4	5	n/a
36.	How often do you avoid attending live sporting events because you can not follow the movement of the players and balls?	0	1	2	3	4	5	n/a
37.	How often have you had to move to a different residence because the world around you seems to move / bounce / jump and you either fell or did not feel safe walking in your home?	0	1	2	3	4	5	n/a
38.	How often do you have trouble sitting and reading a stationary computer screen?	0	1	2	3	4	5	n/a
39.	How often do you have trouble sitting and reading a computer screen while scrolling the screen?	0	1	2	3	4	5	n/a
40.	How often do you have trouble recognizing which light is illuminated on a traffic signal when **driving** a car?	0	1	2	3	4	5	n/a
41.	How often do you have trouble seeing which light is illuminated on a traffic signal when **riding** in a car?	0	1	2	3	4	5	n/a
42.	How often do you have trouble reading something when riding as a passenger in a car?	0	1	2	3	4	5	n/a
43.	How often do you have trouble reading the "ticker" that scrolls on the bottom of the TV when sitting still?	0	1	2	3	4	5	n/a

OFI Total Score___________________

OFI scoring

The OFI Total score is the sum total for all questions.

n/a is scored as 0.

Questions 12, 13, 14, 15, 16, 21, 22, 23, and 24 are phrased negatively, and are scored in reverse order:

0 = 5, 1 = 4, 2 = 3, 3 = 2, 4 = 1, 5 = 0
